# 

**Published:** 1892-01-30

**Authors:** 


PRICE ONE PENNY.

No. 279, Vol. XI.—January 30th, 1892.

[Registered at the General Post Office as a Newspaper for transmission in the United Kingdom and Abroad.]

WITH EXTRA NURSING SUPPLEMENT.
Per Annum, 6s. 6d., post free.
Monthly Parts, 6d.; post free, 8d.
Ditto per Annum, 8s., post free

### THE HOSPITAL

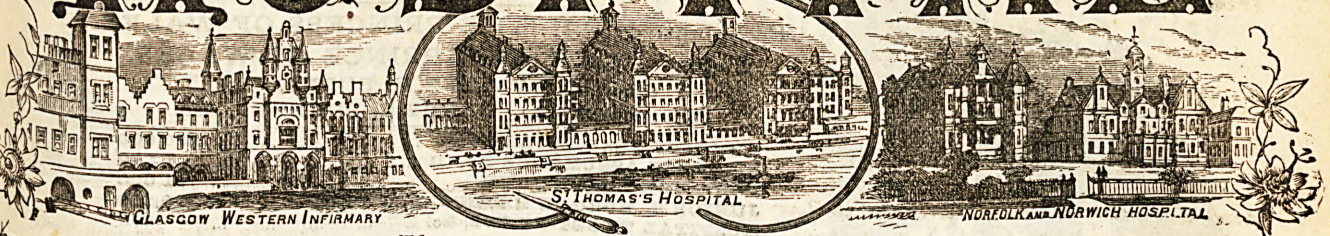

A WEEKLY JOURNAL OF

### Science, Medicine, Nursing, and Philanthropy.

SATURDAY, JANUARY 30th, 1892.

**The Epidemic: Preventive Measures** - 213

**Passing Topics.**—A Reflection - 214

**The Doctor's Armchair.**—No Faith in Physic - 215

**Some Scientific Aspects of Insanity.** X.—Monomania - 216

**Some American Hospitals.** V.—Military Hospitals - 217

**Everybody's Page** - 217

**Notes and News** - 218

**General Charities** - 219

**Editor's Letter-Box.**—"The Man of Genius" - 219

**Scraps and Gleanings** - 219

**Uniform Hospital Accounts** - 223

**Annotations.**—The Risks of Funerals—What next, after Influenza?—Doctors and Lawyers - 220

**The Practitioner's Mirror and Retrospect—**

Medicine.—Venesection in Infantile Diseases - 221

Surgery.—On a New Styptic - 221

Opthalmology.—Phlyctenular Ulceration of the Cornea - 222

New Drugs, Appliances, and Things Medical.—Salipyrin, 222; Messrs. Cadbury and Co.'s Cocoa and Chocolate Productions—New Variety of Eucalyptus Oil - 223

**The Student of Medicine.**—Appointments—Vacancies—Athletics.

EXTRA SUPPLEMENT.—THE NURSING MIRROR.

**En Passant.**—For the Lepers—"We Never Prescribe"—The Nurses' Union—Bedford Institute—New Work for the Blind—Short Items - ciii

**Lectures on Surgical Ward Work and Nursing.** By Alex. Miles, M.D. Edin., F.R.C.S.E.—Lecture XLIII. —Instruments used in Lithotrity - civ

**Lincoln Hospital** - cv

**Presentation** - cv

**For Reading to the Sick.**—Brothers of Pity - cv

**A Canadian Training School** - cvi

**The Nurses' Bookshelf** - cvi

**Everybody's Opinion.**—Hospital Scandals, cvi; On Presentations—The Nurses' Cross—A Vegetarian Dinner - cvii

**Keeping Christmas** - cvii

**Appointments** - cvii

**Off Duty.**—The Little Boy who did not Die in a Hospital - cviii

**Where to Go** - cviii

**Notes and Queries** - cviii

